# Higher Animal-Based Protein Intake Levels Show a Greater Likelihood of Having Metabolic Syndrome in Single-Person Households Among Korean Adults

**DOI:** 10.3390/nu16234239

**Published:** 2024-12-08

**Authors:** Yeongin Lee, Hyojee Joung

**Affiliations:** 1 Institute of Health and Environment, Seoul National University, Seoul 08826, Republic of Korea; dellagem@snu.ac.kr; 2 Department of Public Health, Graduate School of Public Health, Seoul National University, Seoul 08826, Republic of Korea

**Keywords:** single-person households, animal protein, protein source, metabolic syndrome

## Abstract

Background/Objectives: Despite the increasing intake of animal-based protein and the growing number of single-person households (SPHs) in Korean populations, no studies have analyzed the relationship of protein intake by source with metabolic syndrome (MetS) according to household type. This study examined the association between protein intake (plant- and animal-based sources) and MetS risk factors in SPH and multi-person households (MPHs) among Korean adults. Method: A total of 12,022 participants aged 30–64 years (SPH: 982; MPH: 11,040) were selected from the 2016–2021 Korea National Health and Nutrition Examination Survey. Protein intake level was defined as the percentage contribution of food source to daily intake, assessed using 24 h recall dietary data. Results: The animal-based protein intake level was slightly higher in SPHs (51.2%) compared to MPHs (49.5%), whereas the contributions of plant sources from vegetables, fruits, and whole grains were higher in MPHs (*p* < 0.01). The prevalence of MetS and abdominal obesity increased with higher animal-based protein intake levels across all household types. Only in SPHs, each 1% rise in the proportion of animal-based protein was positively associated with increased blood pressure (OR = 1.013, 95% CI: 1.004–1.022). Moreover, the interaction between animal-based protein intake levels and household type was significantly associated with a higher prevalence of elevated triglycerides (TGs) (MPH[Q1] vs. SPH[Q4] OR = 1.51; *p* for interaction = 0.0335). However, these two risk factors did not show significant association in MPHs. Conclusions: The results suggest that reducing dietary animal protein could help manage MetS risk factors, particularly increased blood pressure, and elevated TGs in SPHs of Korean adults. In conclusion, dietary guidelines that promote a higher intake of plant-based protein over animal-based protein for the health of the SPH population would be valuable from a public health perspective.

## 1. Introduction

In 2022, single-person households (SPHs) in Korea accounted for 34.5% of all households, and this figure is projected to continue rising, reaching 39.6% by 2050 [1]. An SPH exhibits certain vulnerable dietary habits compared to an MPH, including a higher rate of meal skipping, more frequent dining out, more irregular mealtimes, and nearly double the rate of nutritional insufficiency [2,3]. Furthermore, adults in SPHs engage in relatively less physical activity and have higher rates of drinking and smoking, along with an increased prevalence of metabolic syndrome (MetS) [4].

MetS is characterized by a cluster of risk factors, including elevated fasting glucose, hypertension, and dyslipidemia, that collectively increase the risk of cardiovascular disease (CVD) and type 2 diabetes [5]. The prevalence of MetS in Korean adults rose from 27.7% before the COVID-19 pandemic to 29.7% after the pandemic, raising public health concerns [6]. Given the increasing prevalence of MetS and the growing SPH population, fundamental strategies for managing MetS, such as lifestyle modifications—including smoking cessation, regular exercise, and proper diet—are necessary for SPH adults in Korea [7].

Protein nutritional status can be assessed based on both quantity and quality. The 2020 Korean Dietary Reference Intakes (KDRIs) established guidelines for protein intake quantity as follows: in grams per body weight (g/kg/day), the estimated average requirement (EAR) is 0.73 g/kg/day, and the recommended nutrient intake (RNI) is 0.91 g/kg/day; and in the percentage of total energy intake (% of energy), the acceptable macronutrient distribution range (AMDR) is 7–20% [8]. Protein intake among Korean adults increased slightly from 14.7% (2010) to 15.6% (2019) of energy, while the proportion of high protein intake (>AMDR) also rose [9]. Protein quality is evaluated by its ability to fulfill the body’s metabolic demands through essential amino acids, which are typically abundant in animal-based foods [10,11]. While 60–70% of total protein intake in Western countries comes from animal sources [12], the proportion of animal-based protein intake in Korea remains lower, with an average of 49.4% across all age groups as of 2021, and a relatively higher share of plant-based protein [13]. However, with the Westernization of the Korean diet, particularly among younger generations [14], the proportion of animal-based protein intake has been rising (aged 19–29: 49.8%, 30–49: 43.9%, 50–64: 36.0% in 2010; 19–29: 56.6%, 30–49: 52.6%, 50–64: 45.9% in 2021) [13].

Previous studies examining the relationship between protein intake (a dietary factor) and MetS have yielded inconsistent evidence. Some research has reported a positive association between protein intake and MetS risk [15,16], with long-term consumption of high-protein diets linked to an increased risk of weight gain, type 2 diabetes, and mortality [17]. Conversely, other studies have found that high-protein diets increase satiety, leading to reduced food intake, which can aid in weight loss and improve cardiovascular markers such as blood lipid levels and blood pressure [18,19]. Nevertheless, it is evident that specific dietary interventions are necessary for protein nutrition, particularly given the complexity of diets and their impact on metabolic health, especially in individuals with metabolic disorders [20].

The growing global population drives higher demand for sustainable protein sources, emphasizing the importance of considering environmental and health contexts specific to each country [21,22]. The discourse on plant-based vs. animal-based protein sources suggests that, from an environmental perspective [22], animal protein consumption may present challenges due to resource usage and greenhouse gas emissions. However, its effects on metabolic health remain unclear, highlighting the need for further research. Recent studies on Korean adults, a cross-sectional study found that higher animal protein intake was associated with a higher prevalence of abdominal obesity, reduced high-density lipoprotein cholesterol (HDL-C), and elevated fasting glucose in men [23]. While a prospective cohort study revealed a negative association between animal protein intake and MetS risk in individuals consuming protein below the RNI [24]. In other countries, some prospective cohort studies have reported the benefits of plant-based protein intake for metabolic disorders [25,26,27], while meta-analyses have found that animal-based protein intake is positively associated with type 2 diabetes [28].

While studies on MetS prevalence in Korean SPH have considered dietary patterns, dietary behaviors, and other health factors as variables, research analyzing the effect of specific protein sources is scarce [4,29,30]. Moreover, previous studies exploring the relationship between protein intake characteristics and metabolic diseases have primarily stratified their analyses by socio-demographic factors such as sex, age, and region; however, none have focused on the effect of household type.

This study was based on the hypothesis that the characteristics of protein intake by food source differ between SPHs and MPHs among Korean adults, leading to different patterns in the association between plant- and animal-based protein intake levels and MetS risk factors. Accordingly, we aimed to highlight the health issues faced by the growing number of SPHs and provide foundational data to inform public health policies and develop appropriate dietary guidelines tailored to SPHs.

## 2. Materials and Methods

### 2.1. Study Population

This study utilized data from the 7th and 8th (2016–2021) Korea National Health and Nutrition Examination Survey (KNHANES), conducted by the Korea Centers for Disease Control and Prevention (KCDC). The KNHANES consists of three examinations (a health interview, a health examination, and a nutrition survey) performed by trained interviewers or medical personnel with the participants’ written consent. The raw data are publicly accessible online https://knhanes.kdca.go.kr/knhanes/sub03/sub03_02_05.do (accessed on 7 December 2024), and detailed information about the study design and protocols is thoroughly described in the website’s guidelines [31]. Of 23,110 adults aged 30–64 years, a total of 12,022 participants were selected after excluding those who were extreme energy-intake outliers (<500 or >5000 kcal/day, n = 3910), pregnant or breastfeeding individuals (n = 251), diagnosed with or receiving treatment for hypertension, dyslipidemia, or diabetes (n = 4970), or had missing data for key variables needed for analysis (n = 1957). Household type was defined based on the household size question in the health interviews: 1 person as single-person households (SPHs, n = 982) and more than 1 person as multi-person households (MPHs, n = 11,040).

### 2.2. Socio-Demographic, Health, and Dietary Behavior Variables

The socio-demographic, health behavior, and dietary behavior variables of the study participants were analyzed using data from the health interview and nutrition survey. Socio-demographic factors included sex, age, education level, household income, and occupational status. Age groups were classified into 30–39, 40–49, and 50–64 years. Education level was categorized as ≤elementary school, middle school, high school, and ≥college/university. Household income was divided into quartiles as lowest, low middle, high middle, and highest. Occupational status was categorized as yes (employed) or no (unemployed or economically inactive).

Health behavior variables included drinking, smoking, physical activity, and health perception. Drinking was assessed using a monthly drinking rate, categorized as <1/month or ≥1/month. Smoking was defined using the current smoking rate, divided by non-smokers and current smokers (those who had smoked at least 100 cigarettes in their lifetime and were currently smoking). Physical activity was determined by the aerobic physical activity rate, classified as active (those who performed at least 2.5 h of moderate-intensity or 1.25 h of high-intensity physical activity per week, or an equivalent combination) and less active (others). Health perception was analyzed through a subjective health perception variable, divided into good, fair, and poor.

Dietary behavior was assessed using breakfast frequency, eating-out frequency, use of nutritional labeling, and food security variables. The breakfast and eating-out frequencies were categorized as ≥3/week and <3/week. Use of nutritional labeling was classified as yes, no, and unsure, while the food security variable was divided into sufficient and insufficient.

### 2.3. Dietary Protein Intake Assessment

Using 24 h dietary recall data collected through face-to-face interviews as part of the nutrition survey, the participants’ energy and nutrient intake were calculated. The adequacy of daily protein intake was assessed by whether intake per body weight met the RNI. The dietary data were coded for each ingredient of foods and dishes, indicating whether it is of plant or animal origin, and the nutrients were calculated based on the intake amount. Protein intake variables were categorized into plant-based and animal-based food sources and further subcategorized by food group and meal type. The plant-based food groups included refined grains products, whole grains products, potato and starches, sugars, legume and legume products, nuts and seeds, vegetables, mushrooms, fruits, seaweeds, condiments, oils, alcohols, and others. The animal-based food groups were pork, beef, poultry, processed meats, other meats, eggs, fish, other seafood, processed seafood, milk and dairy, oils, and others. Meal types were divided into home meals, eating out, conventional foods, catering, and convenience foods.

### 2.4. Diagnostic Criteria of Metabolic Syndromet

Metabolic syndrome was defined by the National Cholesterol Education Program Adult Treatment Panel III (NCEP-ATPIII) criteria as the presence of at least three out of five risk factors: (1) abdominal obesity (waist circumference ≥ 90 cm for men and ≥85 cm for women); (2) increased blood pressure (systolic blood pressure ≥ 130 mmHg or diastolic blood pressure ≥ 85 mmHg); (3) elevated triglycerides (TGs) (TG levels ≥ 150 mg/dL); (4) reduced high-density lipoprotein cholesterol (HDL-C) (HDL-C levels < 40 mg/dL for men and <50 mg/dL for women); (5) elevated fasting glucose (fasting glucose levels ≥ 100 mg/dL) [5,32]. Since waist circumference is recommended to be adjusted to the population characteristics of each country, this study applied the threshold values for Koreans [32]. The anthropometric and clinical measurement data for diagnosing MetS were obtained from the KNHANES health examination.

### 2.5. Statistical Analyses

Statistical analyses were conducted using the Statistical Analysis System (SAS version 9.4, SAS Institute, Cary, NC, USA) program, applying complex sampling design analysis with the variables for cluster (PSU, Primary Sampling Unit), stratification (KSTRATA), and weight (Weight). The statistical significance level was set at α = 0.05 for all analyses.

The distributions of socio-demographic characteristics, health behaviors, dietary behaviors, and the prevalence of MetS and its risk factors were analyzed using the PROC SURVEYFREQ command to compare SPHs and MPHs. Categorical variables were presented with n and %, and statistical significance was tested using the Rao–Scott chi-square test. For comparisons of dietary intakes, the mean and standard error (SE) of continuous variables was presented using a *t*-test (PROC SURVEYREG). The association between protein intake levels and MetS was analyzed using multiple logistic regression analysis (PROC SURVEYLOGISTIC) and estimated odds ratios (ORs) with 95% confidence intervals (95% CIs) for every 1% increase in an independent variable (protein intake level % = protein intake from plant- or animal-based sources/total protein intake × 100).

Potential confounding variables, identified as influencing factors for MetS based on previous studies, such as sex, age, education level, household income, occupational status, drinking, smoking, physical activity, daily energy intake, and protein intake per body weight (RNI fulfillment), were adjusted for in the analysis.

## 3. Results

### 3.1. Characteristics of Study Participants

Table 1 shows the socio-demographic characteristics and MetS prevalence of the participants by household type. The SPH group had a significantly higher proportion of men (65.9%), younger individuals aged 30–39 (43.2%), lower education levels, and a greater percentage with low household income, and were more likely to be occupationally active compared to the MPH group. Among all MetS risk factors, only the prevalence of increased blood pressure (SPH: 30.9%; MPH: 23.0%; *p* < 0.0001) and elevated TGs (SPH: 33.1%; MPH: 28.0%; *p* = 0.0042) were statistically significantly higher in SPHs. However, there were no significant differences by household type in the distribution of MetS or other risk factors such as abdominal obesity, reduced HDL-C, and elevated fasting glucose.

Health and dietary behaviors of study participants are shown in Table 2. SPHs tended to drink and smoke more than MPHs (*p* < 0.01), and a lower proportion perceived their health as good (*p* < 0.0001), with no significant difference in physical activity. Breakfast frequency was lower and the eating-out frequency was higher in SPHs (respectively, *p* < 0.0001). SPHs were also less likely to consume sufficient food (SPH: 96.3%; MPH: 98.9%; *p* < 0.0001).

### 3.2. Nutrient Intakes and Protein Food Sources

The nutrient intake and protein food sources of the participants are presented in Table 3. The absolute amounts of daily intake, such as of energy, carbohydrates, fat, protein, sugar, cholesterol, and sodium, were higher in SPHs than in MPHs (*p* < 0.05). The percentage of energy intake from protein was almost the same between both household types, while carbohydrate intake was lower in SPHs (SPH: 57.0 ± 0.5%; MPH: 59.1 ± 0.2%; *p* = 0.0001), and fat intake was higher in SPHs (SPH: 22.7 ± 0.4; MPH: 21.8 ± 0.1%; *p* = 0.0140). SPHs had a higher intake level of animal-based protein (SPH: 51.2 ± 0.8%; MPH: 49.5 ± 0.2%; *p* = 0.0365) and a relatively lower intake level of plant-based protein (SPH: 48.8 ± 0.8%; MPH: 50.5 ± 0.2%; *p* = 0.0340) compared to MPHs.

For both household types, the primary plant-based protein source was refined grain products (SPH: 25.9 ± 0.6%; MPH: 26.0 ± 0.2%; non-significant difference), followed by vegetables, legumes and legume products, condiments, whole grain products, fruits, and others. Among animal-based food groups, pork was the top source (SPH: 11.8 ± 0.6%; MPH: 11.4 ± 0.2%; *p* = 0.5222), along with poultry, fish, eggs, beef, milk and dairy, and others. Vegetables, whole grains products, and fruits showed significantly higher intake levels in MPHs (*p* < 0.01). Among animal-based sources, SPHs tended to have higher intake levels of poultry, eggs, beef, processed seafood, and processed meat, while MPHs consumed more fish, milk and dairy, and other seafood compared to SPHs. However, these differences were not statistically significant.

**Figure 1:**
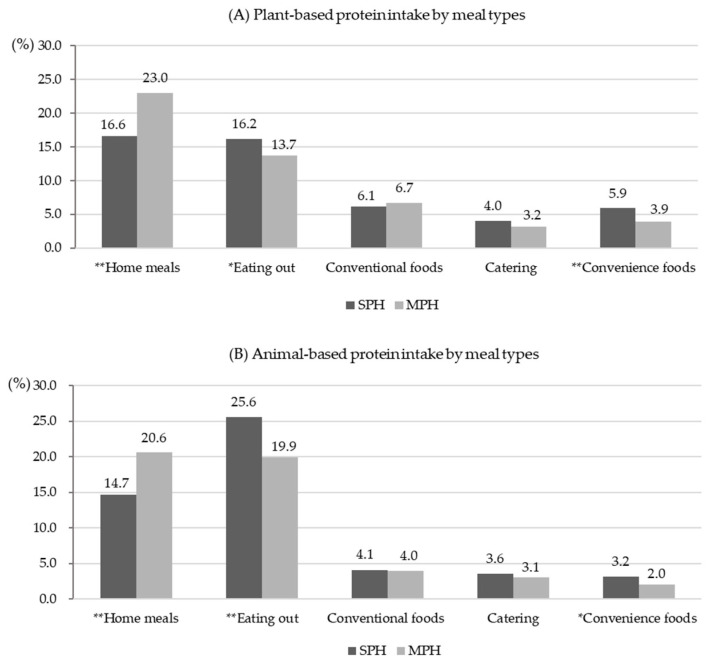
Protein intake level (%) by meal types, stratified by household type. (**A**) Plant-based protein intake by meal types. (**B**) Animal-based protein intake by meal types. *p*-values derived from Student’s *t*-test for continuous variables using PROC SERVEYREG to represent differences between SPHs and MPHs. * *p* < 0.01, ** *p* < 0.0001.

Figure 1 shows the results of plant- and animal-based protein intake levels by meal type. The protein intake level from home meals was significantly higher in MPHs (6.4%p for plant-based sources; 5.9%p for animal-based sources; *p* < 0.0001), while the proportion from eating out was higher in SPHs (2.5%p for plant-based sources, *p* < 0.01; 5.7%p for animal-based sources, *p* < 0.0001). Participants in SPHs consumed a higher proportion of protein from convenience foods compared to MPHs (*p* < 0.01), while there were no significant differences in the protein intake level from conventional foods and catering.

### 3.3. Association of Plant- and Animal-Based Protein Intake Levels with Metabolic Syndrome

Table 4 presents the ORs and 95% CIs for MetS and its risk factors, with each 1% increase in plant- or animal-based protein intake as a proportion of total daily protein intake. Overall, the plant-based protein intake level showed a negative association with the prevalence of MetS risk factors, while the animal-based protein intake level exhibited a positive trend. The association with the animal-based protein intake level was calculated after excluding 66 participants who did not consume animal protein (n = 14 in SPHs; n = 52 in MPHs). The prevalence of MetS and abdominal obesity increased significantly in both household types, with a 1% increase in the animal-based protein intake level. In SPHs, higher levels of animal-based protein intake were associated with a greater prevalence of increased blood pressure (OR = 1.013; 95% CI: 1.004–1.022; *p* < 0.01), whereas no significant trend was observed in MPHs. For elevated fasting glucose, a significant positive association was observed only in MPHs (OR = 1.003; 95% CI: 1.000–1.006; *p* < 0.05). No significant findings were observed for elevated TGs or reduced HDL-C in either household type.

### 3.4. Interaction Between Animal-Based Protein Intake Levels and Household Types on Metabolic Syndrome

The prevalence of MetS risk factors according to the quintiles of animal-based protein intake levels among Korean adults is presented in Table 5. Significant positive associations between the proportion of animal protein intake and both MetS (*p* for trend = 0.0069) and abdominal obesity (*p* for trend < 0.0001) were observed in the total participants. Compared to the lowest quintile group, the highest quintile group of animal-based protein level had a significant higher prevalence of MetS (OR = 1.27; 95% CI: 1.05–1.54; *p* < 0.05) and abdominal obesity (OR = 1.56; 95% CI: 1.31–1.86; *p* < 0.0001).

**Figure 2:**
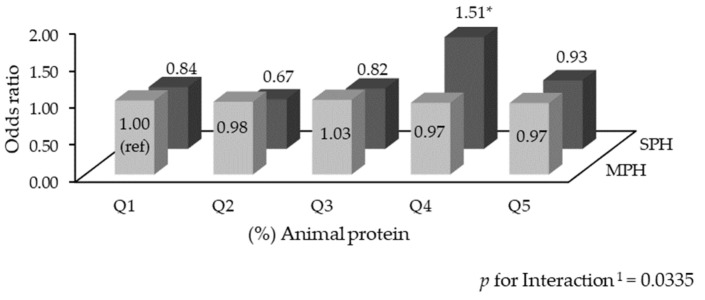
Odds ratios for elevated TGs by quintiles of animal-based protein intake level (%), stratified by household type. Values were calculated from a multivariate logistic regression model (PROC SURVEYLOGISTIC) adjusting sex, age, education level, household income, occupational status, drinking, smoking, physical activity, daily energy intake, and protein intake per body weight (* *p* < 0.05). ^1^ *p* for interaction was derived from a multivariate logistic regression model, presenting the significance of the interaction term between animal-based protein intake level and household type.

To examine the effect of the interaction term between animal protein intake and household type on MetS prevalence, the quintile groups were further stratified by household type. Figure 2 presents the group-specific OR for elevated TGs, which was the only MetS risk factor exhibiting a significant interaction (*p* for interaction = 0.0335). Among SPHs, the group in the fourth quintile of animal-based protein intake showed a higher prevalence compared to the reference group (MPHs in the first quintile), with the interaction term being statistically significant (MPH[Q1] vs. SPH[Q4] OR = 1.51, *p* < 0.05).

## 4. Discussion

This study revealed that the animal-based protein intake level and the prevalence of MetS risk factors differed by household type among 12,022 Korean adults who participated in the 2016–2021 KNHANES. In both household types, animal-based protein intake levels showed a significant positive association with MetS and abdominal obesity. Meanwhile, increased blood pressure and elevated TGs showed a significant increase in prevalence with higher intake of animal-based protein only in SPHs, and elevated fasting glucose exhibiting this trend only in MPHs. Neither SPHs nor MPHs were significantly associated with reduced HDL-C.

In both household types, MetS and abdominal obesity have been reported, as in previous studies, to be favorably affected by plant-based protein intake and adversely affected by animal-based protein intake. A prospective cohort study conducted on adults in Australia found that the % energy from plant protein was negatively associated with the prevalence of MetS and waist circumference (related to abdominal obesity), while the % energy from animal protein showed the opposite effect [33]. In a Belgian population aged 15 years and older, a cross-sectional study reported a negative correlation between waist circumference and plant-based protein intake and a positive association with animal-based protein intake [34]. Red meat consumption was positively associated with abdominal obesity in a longitudinal study on Chinese adults [35]. Similarly, a study using data from the 2013–2018 KNHANES found that middle-aged men with the highest quintile of animal protein intake who had energy intake ≤ EER were more at risk of abdominal obesity compared to the reference group [23]. This study also reported a positive relationship between animal protein intake and the prevalence of reduced HDL-C in men, although no association with reduced HDL-C was observed in our study across both household types [23].

Only in SPHs was there a significant positive association between the animal-based protein intake level and increased blood pressure. Meanwhile, the interaction term between animal-based protein intake levels and household type (the SPH effect) was found to significantly influence the higher prevalence of elevated TGs. The influence of plant- and animal protein intake on these two risk factors has also shown variation in previous studies. A meta-analysis showed that while some observational studies reported an inverse relationship between plant-based protein intake and blood pressure, this was not confirmed in interventional studies, and no association was observed with animal-based protein intake [36]. Another meta-analysis found an inverse relationship between serum TG levels and plant-based protein intake [37]. However, a recent study from Iran, which analyzed FFQ and blood markers from 236 randomly selected participants aged 20–50 from a medical center, reported a higher OR for hypertriglyceridemia in the third tertile compared to the first tertile of plant-based protein intake [38]. A study conducted on Japanese adults observed that the reduction in blood pressure was more strongly associated with an increase in animal-based protein intake than with changes in plant-based protein intake [39]. These inconsistent results suggest that the relationship between plant- or animal-based protein intake and MetS risk factors may vary depending on study methods and populations. Therefore, to establish consistent conclusions, additional high-quality studies across various populations are necessary.

One potential explanation for the differing risk factors associated with plant- and animal-based protein intake levels by household type may be the different characteristics of protein intake. Given the reported association between absolute protein intake and metabolic diseases [24,40], we used the RNI fulfillment status as a covariate to control for intake, allowing us to focus on the influence of protein sources on MetS using the contribution rate of plant- or animal-based protein as an independent variable. Protein sources can affect metabolism in the body through variations in amino acid composition, energy, nutrients (e.g., saturated fatty acids, sugar, cholesterol, sodium, potassium, folate, vitamin C, manganese, and fiber), and bioactive compounds (e.g., phytochemicals) [41,42]. Of these factors, our study adjusted only for energy intake as covariates. Differences in amino acid composition between plant and animal sources may influence the association between protein intake level and metabolic diseases. Non-essential amino acids such as arginine and cysteine are abundant in soy protein, a commonly studied primary source of plant-based protein, and have antihypertensive properties [19]. Lysine and methionine, essential amino acids rich in animal sources, have been shown to induce a significant hypercholesterolemic response [43,44]. Besides amino acids, plant-based foods provide beneficial components like dietary fiber, antioxidants, and phytochemicals, while animal sources are relatively higher in saturated fats and cholesterol [33]. Excessive intake of saturated fats and cholesterol can affect serum lipid levels and potentially lead to coronary heart disease [45,46,47], so greater caution is needed when consuming animal-based foods compared to plant-based foods. Considering that increased intake of each protein source can lead to higher intake of associated nutrients, along with our study results, we may recommend moderating animal-based protein intake to reduce metabolic risk. However, excessively low intake of animal-based protein may lead to deficiencies in micronutrients such as iron and vitamin B12; therefore, it is advisable to refer to the recommended range of 15–80% for the plant-based protein intake ratio [48].

Another possible reason for the differences in prevalence patterns between household types interacting with animal protein intake could be dietary factors, including other nutrient intakes, breakfast frequency, and eating-out behavior. First, SPHs tend to have higher daily intakes of sodium, carbohydrates, and sugar according to this study. Therefore, careful attention to sodium intake, high-carbohydrate diets, and sugary drinks may be needed, as these factors can contribute to increased blood pressure and elevated TG levels [49,50]. Secondly, SPHs ate breakfast less frequently than MPHs among our study participants. Skipping breakfast can lower the basal metabolic rate, and overeating after a prolonged fasting period can lead to a rapid increase in blood glucose levels and promote fat synthesis, resulting in fat accumulation in the body [51]. In a longitudinal study from Australia, individuals who skipped breakfast both in childhood and adulthood were found to have a larger waist circumference, higher fasting insulin, and total cholesterol concentrations, thereby increasing their risk of cardiovascular disease compared to those who ate breakfast at both time points [51]. A prospective cohort study tracking 3598 U.S. adults found that participants who ate breakfast daily had significantly lower hazard ratios (HRs) for MetS, abdominal obesity, and hypertension compared to those who ate breakfast ≤ 3/week [52]. In studies on Korean adults, while some findings have indicated a relatively higher prevalence of MetS among those who skipped breakfast [53], others have shown that individuals who rarely ate breakfast had lower daily energy intake and a relatively lower risk of elevated TGs compared to regular breakfast eaters [54]. Despite these conflicting results, acknowledging that breakfast frequency affects MetS and its risk factors justifies its use as a covariate. Lastly, we found that eating-out frequency was higher in SPHs than in MPHs, with distinct characteristics in protein intake levels by meal type. SPHs had the highest proportion of animal-based protein intake from eating-out (25.6%), while MPHs had the highest proportion of plant-based protein intake from home meals (23.0%). Studies using KNHANES data have shown that eating-out diets tend to have higher intakes of energy, fat, cholesterol, and sodium, a higher proportion of saturated fatty acids, and lower dietary fiber intake compared to home meals, indicating a positive association between an increased frequency of eating out and the risk of chronic diseases [53,55,56]. Although the eating-out frequency was not considered a confounding factor in our study, previous research and the higher eating-out frequency in SPHs compared to MPHs suggest that it would be beneficial to establish guidelines for healthy dining out tailored to SPH dietary habits.

Health behaviors also demonstrate different patterns between SPHs and MPHs, which need to be addressed. In our study, the proportion of individuals who drink and smoke was higher in SPHs, and fewer people reported their health as good compared to MPHs. Previous studies have reported higher prevalence of MetS in groups that engage in drinking and smoking [6,15], while that of those who rated their health as poor or bad was significantly higher in the MetS group compared to the normal group [57]. Therefore, when implementing public health programs aimed at preventing metabolic syndrome in SPHs, improving health behavior indicators such as drinking, smoking, and health perception should also be considered.

This study had the following limitations. First, due to the cross-sectional nature of the data, it is difficult to clearly explain the causal relationship between protein intake levels and MetS. Secondly, the use of a single day of 24 h recall method data in this study may have led to random and systematic errors, producing bias and resulting in inaccurate information about an individual’s usual intake of nutrients and foods. Thirdly, we did not explore the correlations between protein intake levels from meal types or food groups, just categorizing the variables into plant and animal-based groups. In order to provide concrete evidence for dietary guidelines aimed at preventing MetS, further analysis is needed on the relationship between detailed protein sources and the prevalence of MetS. Fourth, this study did not clarify how certain health factors associated with each household type interact with protein intake levels to produce different patterns of ORs for increased blood pressure, elevated TGs, and elevated fasting glucose.

To address these issues, longitudinal data or intervention studies could be used to support or strengthen the evidence on the associations or causal relationships found in this study. Using more than 2 days of 24 h dietary recall data or analyzing larger populations may reduce daily dietary variation and more clearly identify the relationship between individual variation in protein intake and MetS. In addition, amino acid databases are needed for evaluating the health effects of protein quality and reinforcing the biochemical mechanisms involved. It would be useful to analyze the effect of health and dietary behavior variables or the intake of other nutrients, which were not considered as covariates, to identify lifestyle habits that should be improved alongside protein sources in SPHs.

Despite these limitations, this study is significant as it is the first to investigate the distribution of protein intake sources and levels among SPHs and MPHs in Korean adults and their associations with MetS and its risk factors.

## 5. Conclusions

MetS and abdominal obesity were more prevalent among individuals with higher animal-based protein intake levels across all household types. Increased blood pressure was positively associated with animal-based protein intake levels only in SPHs, while elevated fasting glucose showed the same effect only in MPHs. Interaction between animal protein intake and household types resulted in higher prevalence of elevated TGs among SPHs. No significant associations were found for reduced HDL-C. These results suggest that moderating animal-based protein intake levels in SPH adults in Korea may be a potential strategy for managing blood pressure and triglyceride levels. Public health programs aimed at preventing MetS in SPHs could be more effective by substituting animal-based proteins with plant-based proteins, providing healthy eating out guidelines, and promoting alcohol reduction and smoking cessation. However, longitudinal studies are needed to establish causal relationships and address the limitations of this cross-sectional study.

## Figures and Tables

**Table 1:** Socio-demographic factors and metabolic syndrome prevalence of the participants by household type.

n (%)	Total (n = 12,022)	SPH ^2^ (n = 982)	MPH ^3^ (n = 11,040)	*p*-Value ^1^
**Demographic factors**				
Sex				<0.0001
Males	4747 (49.0)	516 (65.9)	4231 (47.5)	
Females	7275 (51.0)	466 (34.1)	6809 (52.5)	
Age				<0.0001
30–39 years	3617 (33.3)	317 (43.2)	3300 (32.5)	
40–49 years	4129 (34.7)	241 (25.6)	3888 (35.4)	
50–64 years	4276 (32.0)	424 (31.2)	3852 (32.1)	
Education level				0.0030
≤Elementary school	549 (3.5)	81 (4.9)	468 (3.3)	
Middle school	808 (5.7)	107 (7.6)	701 (5.5)	
High school	4043 (33.3)	333 (32.5)	3710 (33.3)	
≥College/university	6622 (57.6)	461 (55.0)	6161 (57.9)	
Household income				<0.0001
Lowest	801 (6.1)	199 (17.7)	602 (5.1)	
Low middle	2751 (21.9)	250 (21.8)	2501 (21.9)	
High middle	3930 (33.1)	278 (31.8)	3652 (33.2)	
Highest	4540 (38.9)	255 (28.7)	4285 (39.8)	
Occupational status				<0.0001
Yes	8750 (75.0)	768 (80.6)	7982 (74.5)	
No	3272 (25.0)	214 (19.4)	3058 (25.5)	
**MetS ^4^ and risk factors**				
Metabolic syndrome	2296 (20.3)	214 (22.2)	2082 (20.2)	0.1996
Abdominal obesity	2988 (26.3)	253 (28.0)	2735 (26.2)	0.2835
Increased blood pressure	2714 (23.7)	291 (30.9)	2423 (23.0)	<0.0001
Elevated TGs ^5^	3159 (28.4)	300 (33.1)	2859 (28.0)	0.0042
Reduced HDL-C ^6^	3601 (28.8)	273 (26.0)	3328 (29.0)	0.0846
Elevated fasting glucose	3348 (28.7)	293 (29.9)	3055 (28.6)	0.4583

^1^ *p*-values derived from Rao–Scott chi-squared test for categorical variables using PROC SURVEYFREQ to represent differences between SPHs and MPHs. ^2^ SPH = single-person household. ^3^ MPH = multi-person household. ^4^ MetS = metabolic syndrome. ^5^ TG = triglyceride. ^6^ HDL-C = high-density lipoprotein cholesterol.

**Table 2:** Health and dietary behaviors of the study participants by household type.

n (%)	Total (n = 12,022)	SPH (n = 982)	MPH (n = 11,040)	*p*-Value ^1^
**Health behaviors**				
Drinking				0.0001
<1/month	4953 (38.6)	373 (32.5)	4580 (39.1)	
≥1/month	7069 (61.4)	609 (67.5)	6460 (60.9)	
Smoking				<0.0001
Non-smoker	9870 (78.9)	672 (63.7)	9198 (80.2)	
Current smoker	2152 (21.1)	310 (36.3)	1842 (19.8)	
Physical activity				0.7403
Active	5435 (46.7)	440 (47.3)	4995 (46.6)	
Less active	6587 (53.3)	542 (52.7)	6045 (53.4)	
Health perception				<0.0001
Good	4165 (35.0)	281 (30.2)	3884 (35.4)	
Fair	6458 (53.8)	545 (53.9)	5913 (53.8)	
Poor	1399 (11.2)	156 (15.9)	1243 (10.8)	
**Dietary behaviors**				
Breakfast				<0.0001
≥3/week	8145 (64.8)	559 (50.1)	7586 (66.1)	
<3/week	3877 (35.2)	423 (49.9)	3454 (33.9)	
Eating out				<0.0001
≥3/week	7251 (64.1)	682 (76.5)	6569 (63.0)	
<3/week	4771 (35.9)	300 (23.5)	4471 (37.0)	
Use of nutritional labeling				0.0592
Yes	4296 (35.0)	321 (33.9)	3975 (35.1)	
No	6324 (53.8)	502 (52.1)	5822 (53.9)	
Unaware	1402 (11.2)	159 (14.0)	1243 (11.0)	
Food security				<0.0001
Sufficient	11,844 (98.7)	934 (96.3)	10,910 (98.9)	
Insufficient	178 (1.3)	48 (3.7)	130 (1.1)	
Protein intake ^2^				0.0932
<0.91 g/kg/day	4466 (36.8)	342 (34.0)	4124 (37.0)	
≥0.91 g/kg/day	7556 (63.2)	640 (66.0)	6916 (63.0)	

^1^ *p*-values derived from Rao–Scott chi-squared test for categorical variables using PROC SURVEYFREQ to represent differences between SPHs and MPHs. ^2^ Protein intake = Protein intake per body weight (recommended nutrient intake, RNI: 0.91 g/kg/day).

**Table 3:** Nutrient intakes and protein sources of the study participants by household type.

Mean ± SE	SPH (n = 982)	MPH (n = 11,040)	*p*-Value ^1^
**Daily intake**			
Energy (kcal)	2166.1 ± 33.3	1998.8 ± 9.9	<0.0001
Carbohydrate (g)	297.7 ± 4.4	287.2 ± 1.4	0.0180
Fat (g)	56.8 ± 1.5	49.5 ± 0.4	<0.0001
Protein (g)	81.2 ± 1.6	74.5 ± 0.4	<0.0001
Sugar (g)	67.7 ± 1.6	62.7 ± 0.5	0.0022
Cholesterol (mg)	297.9 ± 10.9	268.0 ± 2.5	0.0062
Sodium (mg)	3733.9 ± 79.0	3507.3 ± 24.5	0.0042
**% Energy from Nutrients**			
Carbohydrate	57.0 ± 0.5	59.1 ± 0.2	0.0001
Fat	22.7 ± 0.4	21.8 ± 0.1	0.0140
Protein	15.0 ± 0.2	15.0 ± 0.1	0.7723
**% Protein sources ^2^**			
Plant protein	48.8 ± 0.8	50.5 ± 0.2	0.0340
Refined grain products	25.9 ± 0.6	26.0 ± 0.2	0.8647
Vegetables	6.7 ± 0.2	7.3 ± 0.1	0.0006
Legume and legume products	4.8 ± 0.3	4.9 ± 0.1	0.5992
Condiments	3.4 ± 0.1	3.4 ± 0.0	0.6622
Whole grain products	1.7 ± 0.2	2.3 ± 0.1	0.0003
Fruits	1.2 ± 0.1	1.6 ± 0.0	<0.0001
Nuts and seeds	1.1 ± 0.1	1.2 ± 0.0	0.5722
Animal protein	51.2 ± 0.8	49.5 ± 0.2	0.0365
Pork	11.8 ± 0.6	11.4 ± 0.2	0.5222
Poultry	7.5 ± 0.7	6.8 ± 0.2	0.2993
Fishes	6.6 ± 0.5	6.7 ± 0.1	0.8898
Eggs	6.6 ± 0.4	6.4 ± 0.1	0.6012
Beef	5.9 ± 0.5	5.8 ± 0.1	0.8788
Milk and dairy	4.1 ± 0.3	4.3 ± 0.1	0.6242
Other seafoods	3.9 ± 0.4	4.0 ± 0.1	0.7382
Processed seafoods	2.3 ± 0.3	2.2 ± 0.1	0.6246
Processed meat	2.3 ± 0.2	1.9 ± 0.1	0.0837

^1^ *p*-values derived from Student’s *t*-test for continuous variables using PROC SERVEYREG to represent differences between SPHs and MPHs. ^2^ % Protein sources = Protein intake from each source/Total protein intake × 100.

**Table 4:** Odds ratios and 95% confidence intervals for metabolic syndrome by household type, with each 1% increase in plant- and animal-based protein intake level (%).

% Plant Protein	Total (n = 12,022)	SPH (n = 982)	MPH (n = 11,040)
Metabolic syndrome	0.995 (0.992–0.998) **	0.984 (0.974–0.994) **	0.996 (0.993–0.999) *
Abdominal obesity	0.991 (0.988–0.994) ***	0.985 (0.975–0.995) **	0.992 (0.989–0.995) ***
Increased blood pressure	0.997 (0.994–1.000) *	0.987 (0.979–0.996) **	0.998 (0.994–1.001)
Elevated TGs	1.000 (0.997–1.002)	0.991 (0.983–1.001)	1.001 (0.998–1.003)
Reduced HDL-C	0.999 (0.997–1.002)	0.992 (0.982–1.001)	1.000 (0.997–1.003)
Elevated fasting glucose	0.997 (0.994–0.999) *	0.996 (0.987–1.005)	0.997 (0.994–1.000) *
**% Animal protein**	**Total (n = 11,956)**	**SPH (n = 968)**	**MPH (n = 10,988)**
Metabolic syndrome	1.005 (1.002–1.008) **	1.017 (1.006–1.027) **	1.004 (1.001–1.007) *
Abdominal obesity	1.009 (1.006–1.012) ***	1.015 (1.005–1.026) **	1.008 (1.005–1.012) ***
Increased blood pressure	1.003 (1.000–1.007) *	1.013 (1.004–1.022) **	1.002 (0.999–1.006)
Elevated TGs	1.000 (0.998–1.003)	1.008 (0.999–1.017)	0.999 (0.997–1.002)
Reduced HDL-C	1.001 (0.998–1.003)	1.008 (0.999–1.018)	1.000 (0.997–1.003)
Elevated fasting glucose	1.004 (1.001–1.006) *	1.004 (0.995–1.013)	1.003 (1.000–1.006) *

Values were calculated from a multivariate logistic regression model (PROC SURVEYLOGISTIC) adjusting sex, age, education level, household income, occupational status, drinking, smoking, physical activity, daily energy intake, and protein intake per body weight (* *p* < 0.05, ** *p* < 0.01, *** *p* < 0.0001).

**Table 5:** Odds ratios and 95% confidence intervals for metabolic syndrome according to quintiles of animal-based protein intake level (%) in total participants.

% Animal Protein	Q1	Q2	Q3	Q4	Q5	*p* for Trend ^1^
Total (n = 11,956)	2391	2391	2392	2391	2391	
SPH (n = 968)	203	208	155	197	205	
MPH (n = 10,988)	2188	2183	2237	2194	2186	
Median intake (%) ^2^	23.6 ± 0.3	39.1 ± 0.2	50.2 ± 0.1	60.3 ± 0.1	73.0 ± 0.2	
Metabolic syndrome	1.00 (ref)	1.00 (0.84–1.18)	1.27 (1.06–1.52) **	1.24 (1.03–1.48) *	1.27 (1.05–1.54) *	0.0069
Abdominal obesity		1.02 (0.86–1.20)	1.40 (1.19–1.66) ***	1.38 (1.17–1.63) **	1.56 (1.31–1.86) ***	<0.0001
Increased blood pressure		0.97 (0.82–1.14)	1.12 (0.95–1.32)	1.12 (0.94–1.34) *	1.16 (0.97–1.39)	0.1584
Elevated TGs		0.97 (0.83–1.13)	1.01 (0.86–1.19)	1.02 (0.87–1.21)	0.98 (0.83–1.15)	0.9431
Reduced HDL-C		0.85 (0.73–0.98) *	0.94 (0.82–1.09)	0.95 (0.82–1.10)	1.02 (0.88–1.19)	0.0893
Elevated fasting glucose		1.10 (0.94–1.29)	1.18 (1.01–1.38) *	1.19 (1.01–1.39) *	1.22 (1.04–1.44) *	0.1224

Values were calculated from a multivariate logistic regression model (PROC SURVEYLOGISTIC) adjusting sex, age, education level, household income, occupational status, drinking, smoking, physical activity, daily energy intake, and protein intake per body weight (* *p* < 0.05, ** *p* < 0.01, *** *p* < 0.0001). ^1^ *p* for the trend was derived from a multivariate logistic regression model using the median intake (%) for each quintile as the continuous variable. ^2^ Median intake (%) = median value of animal-based protein intake level (mean ± SE).

## Data Availability

The data presented in this study are available on request from the corresponding author.
